# Loneliness and Schizotypy Are Distinct Constructs, Separate from General Psychopathology

**DOI:** 10.3389/fpsyg.2016.01018

**Published:** 2016-07-07

**Authors:** Johanna C. Badcock, Emma Barkus, Alex S. Cohen, Romola Bucks, David R. Badcock

**Affiliations:** ^1^Centre for Clinical Research in Neuropsychiatry, School of Psychiatry and Clinical Neurosciences, The University of Western AustraliaPerth, WA, Australia; ^2^School of Psychology, University of WollongongWollongong, NSW, Australia; ^3^Department of Psychology, Lousiana State UniversityBaton Rouge, LA, USA; ^4^School of Psychology, The University of Western AustraliaCrawley, WA, Australia

**Keywords:** loneliness, schizotypal traits, bi-factor model, psychopathology, psychosis continuum

## Abstract

Loneliness is common in youth and associated with a significantly increased risk of psychological disorders. Although loneliness is strongly associated with psychosis, its relationship with psychosis proneness is unclear. Our aim in this paper was to test the hypothesis that loneliness and schizotypal traits, conveying risk for schizophrenia spectrum disorders, are similar but separate constructs. Pooling data from two non-clinical student samples (*N* = 551) we modeled the structure of the relationship between loneliness and trait schizotypy. Loneliness was assessed with the University of California, Los Angeles Loneliness Scale (UCLA-3), whilst negative (Social Anhedonia) and positive (Perceptual Aberrations) schizotypal traits were assessed with the Wisconsin Schizotypy Scales-Brief (WSS-B). Fit statistics indicated that the best fitting model of UCLA-3 scores comprises three correlated factors (Isolation, Related Connectedness, and Collective Connectedness), consistent with previous reports. Fit statistics for a two factor model of positive and negative schizotypy were excellent. Next, bi-factor analysis was used to model a general psychopatholgy factor (p) across the three loneliness factors and separate negative and positive schizotypy traits. The results showed that all items (except 1) co-loaded on p. However, with the influence of p removed, additional variance remained within separate sub-factors, indicating that loneliness and negative and positive trait schizotypy are distinct and separable constructs. Similarly, once shared variance with p was removed, correlations between sub-factors of loneliness and schizotypal traits were non-significant. These findings have important clinical implications since they suggest that loneliness should not be conflated with the expression of schizotypy. Rather, loneliness needs to be specifically targeted for assessment and treatment in youth at risk for psychosis.

## Introduction

Loneliness is common in adolescents and young adults, involving perceived (as opposed to objective) isolation from others (Rönkä et al., 2014; Qualter et al., 2015). Higher levels of loneliness are associated with a reduction in well-being and an increased risk for various forms of psychopathology, though much of the prior research has focused on the link with depression (Lasgaard et al., 2011; Vanhalst et al., 2012; Shevlin et al., 2014). Recently there has been a growing interest in the relationship between loneliness and psychosis (Stain et al., 2012; Sundermann et al., 2014) with 75–94% of people with psychotic disorders reporting feeling lonely some or more of the time (Badcock et al., 2015). However, the specific relationship between loneliness and psychosis proneness remains unclear (Lim and Gleeson, 2014).

From a clinical perspective, high trait schizotypy is clearly associated with an increased risk of schizophrenia-spectrum psychopathology (Barrantes-Vidal et al., 2013; Cicero et al., 2014). The term “schizotypy” (a contraction of schizophrenic phenotype) describes a cluster of personality traits that convey vulnerability to psychosis, including unusual perceptual experiences, magical thinking, and paranoid ideas (positive schizotypy) and constricted affect, anhedonia, and social anxiety (negative schizotypy). These characteristics broadly correspond to the positive (hallucinations, delusions) and negative (anhedonia, flattened affect) symptom dimensions of schizophrenia. The Wisconsin Schizotypy Scales (WSS) are widely used to assess schizotypal traits and confirmatory factor analysis indicates that a two-factor structure, composed of positive and negative schizotypy, underpins these scales (Gross et al., 2014, 2015). Furthermore, high schizotypy scores are correlated with psychotic-like, prodromal and schizophrenia-spectrum symptoms, as well as increased risk for developing psychosis in the future (e.g., Barrantes-Vidal et al., 2013; Cicero et al., 2014). However, the boundaries between loneliness and schizotypal traits may be difficult to discern (Nelson et al., 2013; Cohen et al., 2015). For example, it is unclear if loneliness is a consequence, or a core component, of positive schizotypy (e.g., unusual perceptual experiences, persecutory beliefs), given that paranoia and suspiciousness result in social distancing and/or withdrawal (Cohen et al., 2015). Alternatively, the conceptual overlap may be most evident on measures of negative schizotypy (e.g., social anhedonia, lack of pleasure), which often include items about feeling lonely and socially disconnected (e.g., WSS item 7: “I dont really feel very close to my friends”; Chapman et al., 1976; Gross et al., 2012). Therefore, loneliness and schizotypy may easily be conflated and treated as the same construct, i.e., as evidence of generalized psychopathology, reflecting a common liability to mental disorder (Caspi et al., 2014). However, if loneliness and schizotypy are similar but separable experiences, then the clinical consequences of assuming they are equivalent could be significant: for example, leading loneliness to be missed or mistaken for social anhedonia or a facet of paranoid thinking, rather than an independent problem with separate functional effects and treatment needs in people vulnerable to psychosis.

From a theoretical and empirical point of view, loneliness is generally recognized as being multi-dimensional in nature (Hawkley et al., 2005; Durak and Senol-Durak, 2010; Shevlin et al., 2015). These findings typically indicate that our mental map of the social world involves individual, interpersonal and collective aspects, each with different causes and consequences for social functioning (Hawkley et al., 2012). For example, Shevlin et al. (2015) investigated the multidimensional structure of the widely-used UCLA Loneliness Scale, version 3 (UCLA-3; Russell, 1996) using confirmatory factor analysis, and found that the best fitting model comprised three correlated factors. The first factor, termed Isolation, included items tapping feelings of being alone and lacking companionship. The second, Relational Connectedness, factor reflected more social aspects of loneliness, such as feeling there are other people one can turn to or talk to. Finally, the third factor, termed Collective Connectedness, consisted of items such as feeling in tune with others and part of a group of friends, and appears to relate to the sense of connection to a group or community. Importantly, poor social functioning has been associated with both negative and positive schizotypy, adversely impacting on quality of life (Cohen and Davis, 2009; Abbott et al., 2012). However, there have been no previous studies examining whether these individual, interpersonal, and collective factors of loneliness are differentially related to positive and negative schizotypy traits, which would bolster the utility of a multi-dimensional loneliness model.

Motivated by these clinical and theoretical issues, the goal of the current study was to determine if loneliness and schizotypal traits are distinct and separable constructs, or whether they are all aspects of a single, common dimension: general psychopathology (the “p” factor; Caspi et al., 2014).

## Materials and methods

### Participants

Participants comprised a convenience sample of 551 students (*N* = 381 females; 69.1%), from two separate studies conducted at the University of Western Australia (UWA; *N* = 246) and the University of Wollongong (UoW; *N* = 305). The age range of the sample was 15–55 years, though the majority (90.7%) were young adults aged under 25 (Mean age = 20.6, *SD* = 5.53).

### Measures

#### Assessment of loneliness

The UCLA Loneliness Scale, version 3 (Russell, 1996) is a brief and well-established measure of loneliness, with good psychometric properties (UCLA-3; Russell, 1996; Lasgaard, 2007; Vassar and Crosby, 2008). Participants are presented with an introductory statement: “How often do you….” followed by 20 items assessing how people perceive their social situation without explicitly using the terms “lonely” or “loneliness: (e.g., ….feel part of a group of friends). Each item is rated on a 4-point Likert-type scale, reflecting increasing feelings on loneliness: 1 (*Never*); 2 (*Rarely*); 3 (*Sometimes*); 4 (*Often*). After reverse scoring of relevant items, responses are summed, yielding a maximum total score of 80.

#### Assessment of schizotypal personality traits

Positive and negative schizotypal traits were assessed with the Perceptual Aberration (PAb) and Social Anhedonia (SA) scales from the Wisconsin Schizotypy Scales-Brief (WSS-B) (Gross et al., 2012)^1^. Previous analyses indicate that these scales tap independent dimensions of the schizotypy construct, and are significantly correlated with interview measures of psychotic-like symptoms (Gross et al., 2012; Fonseca-Pedrero et al., 2013). These self-report scales containing 15 items each, scored True/False, and were interleaved together. In the UWA sample, the WSS-B were also interspersed with infrequency items (Chapman and Chapman, 1983) designed to detect random or careless responding (e.g*., I cannot remember a single occasion when I have ridden on a bus*).

### Procedure

Participants in the UWA sample were enrolled in a first year “broadening unit” in psychology (comprising students from a variety of arts and science majors). They completed a series of questionnaires in supervised groups, following an introductory class on research methods, and were offered course credit for completing the session. Those with three or more positive responses on the infrequency scale were eliminated from the final sample, *N* = 4, hence data from 242 students remained for further analyses. Participants in the UoW sample were primary recruited through a research participation pool which all first years and a section of second year psychology students complete for course credit. Other individuals were recruited through snowballing. Participants were eliminated from the final sample if they had not completed all measures, *n* = 5, and if there was no variability in their responses indicating they had not engaged with the questions *n* = 3. All participation was voluntary and human research ethics approval was obtained from the relevant institutional review board at each study site.

### Data analysis

Analyses were conducted using IBM SPSS Statistics 22 (IBM, 2013) and Mplus 7.31 (Muthén and Muthén, 2010). To test the hypothesis that loneliness is multi-dimensional and to generalize existing factor structures to Australian samples, in Phase 1 of the analyses (*N* = 551), traditional confirmatory factor analysis was employed to test the fit of the UCLA-3 data to three, alternative models. One-factor (labeled “Loneliness”; Russell, 1996), two-factor (labeled “Intimate Other” and “Social Other”; Wilson et al., 1992) and three-factor models (labeled “Isolation,” “Relational Connectedness,” and “Collective Connectedness;” Hawkley et al., 2005) have previously been described, with the latter (comprising correlated factors) considered the best fitting model in adolescent (Shevlin et al., 2015) and adult samples (Hawkley et al., 2005, 2012).

The next step, Phase 2, was to check the factor structure of the schizotypy data using a 2 factor (i.e., a positive and negative schizotypy) model.

Phase 3, then combined the best fitting UCLA-3 model with the 2 factor positive and negative schizotypy model (*N* = 242) to test the hypothesis that sub-factors of loneliness and schizotypy exist outside a general psychopathology factor (designated as “p”). Traditional, confirmatory factor analytic techniques assume that each item loads onto a single dimension. However, questionnaire items are rarely indicators of a single construct. Rather, they may be multidimensional, sharing a degree of association with other constructs (Reise, 2012; Morin et al., 2016). Confirmatory bi-factor models are one set of models gaining traction as a technique for accounting for this multidimensionality (Reise, 2012). A bi-factor model partials out covariance that is shared by all scale items into a single “general” psychopathology factor (“p”) which reflects individual differences in what is common amongst the items, whilst simultaneously identifying two or more orthogonal sub-factors, or item parcels, representing common factors shared by those items that explain variance not accounted for by the general factor. By using bi-factor modeling, we were able to test the hypothesis that the three facets of loneliness and separate positive and negative schizotypy traits existed outside of a single, general psychopathology factor.

The final step, in Phase 4, was to explore the pattern of associations between the sub-factors of loneliness and positive and negative schizotypy traits, once the variance attributable to general psychopathology was removed, to determine any differential relationships that exists.

In all analyses, a weighted least squares mean and variance adjusted (WLSMV) estimator was used. This robust estimator was developed for use with categorical or ordinal data, and was designed for use with polychoric correlations (Muthen et al., unpublished data^2^). Model fit was examined with a range of fit statistics. Given that chi-square is highly sensitive to sample size (Marsh et al., 1988), we also report the comparative fit index (CFI: Bentler, 1990) and the Tucker–Lewis index (TLI: Tucker and Lewis, 1973) where values above 0.90 indicated reasonable fit, and above 0.95 good fit. The TLI compares the fit of each theoretically derived model to a null or baseline model, which assumes no relationships between the variables. This index is less affected by sample size than chi-square (Marsh et al., 1988) and is, therefore, useful for comparing factor models. For the root mean square error of approximation (RMSEA; Steiger, 1990) values < 0.05 indicate good fit, and values from 0.05 to 0.10 suggest moderate fit (Bentler, 1990; Hu et al., 1999. Finally, following Thompson (1997), pattern and structure coefficients were used to determine whether constructs in measurement models were empirically distinguishable (i.e., to assess discriminant validity). Pattern coefficients are the standardized factor loadings. To determine the structure coefficients, the influence of each factor on items not hypothesized to comprise that factor is calculated by multiplying the latent factor correlation by the factor loadings of the item.

## Results

The average for the UCLA-3 total score was 42.62 (*SD* = 10.69, range = 20–71). The mean PAb score was 1.02 (*SD* = 2.22, range = 0–14) and for the SA score was 2.63 (*SD* = 2.88, range = 0–15).

### Phase 1: Confirmatory factor analysis of UCLA-3 data

Fit statistics for the three UCLA-3 models are shown in Table 1. Both the two and three-factor models showed good fit based on the CFI and TLI, and either would be acceptable. Factor loadings for the one, two and three-factor models are shown in Table 2, all of which were statistically significant (*p* < 0.05). Factor inter-correlations were also strong and significant for both the 2 factor (Intimate Other with Social Other *r* = 0.80, *p* < 0.001) and 3 factor models (Related Connectedness with Isolation, *r* = 0.78, *p* < 0.001; Collective Connectedness with Isolation, *r* = 0.79, *p* < 0.001; Collective Connectedness with Relational Connectedness, *r* = 0.86, *p* < 0.001). Although the RMSEA for the 3-factor model was marginally lower at 0.08, it has previously been found to be the best fitting model in prior research, as noted above (Shevlin et al., 2015), so we opted to take the 3-factor model into the next phase of analysis. Moreover, inspection of the indirect loadings for the 3-factor model revealed that these never exceeded the strength of the direct loadings, suggesting good discriminant validity (Thompson, 1997; see Supplementary Table 1 for pattern and structure matrices for the UCLA-3).

**Table 1:** **Confirmatory and bi-factor models of the UCLA-3 Loneliness Scale and WSS-B Perceptual Aberration and Social Anhedonia scales, with fit statistics**.

**No**.	**Model**	**χ^2^**	***Df***	***p***	**CFI**	**TLI**	**RMSEA**	**95% CI**
**PHASE 1 LONELINESS MODELS**								
1	1 factor (Russell, 1996)	1598.86	170	< 0.001	0.92	0.91	0.12	0.12–0.13
2	2 factor correlated (Wilson et al., 1992)	843.14	169	< 0.001	0.96	0.96	0.09	0.08–0.09
3	3 factor correlated (Hawkley et al., 2005)	809.48	167	< 0.001	0.96	0.96	0.08	0.08–0.09
**PHASE 2 SCHIZOTYPY MODEL**								
4	2 factor correlated (positive and negative schizotypy)	400.26	349	0.030	0.97	0.97	0.02	0.01–0.02
**PHASE 3 BI-FACTOR ORTHOGONAL MODEL**								
5	With “p” and 5 sub-factors (Isolation, Relational Connectedness, Collective Connectedness, Positive Schizotypy, Negative Schizotypy)	1339.29	1032	< 0.001	0.98	0.98	0.02	0.02–0.03
**PHASE 4 BI-FACTOR MODEL, EXPLORING, CORRELATIONS**								
6	With associations between the loneliness and schizotypy sub-factors in the absence of “p”	1363.86	1026	< 0.001	0.98	0.98	0.02	0.02–0.03

*CFI, comparative fit index, TLI, Tucker-Lewis index, RMSEA, root mean square error of approximation, 95% CI for RMSEA, confidence interval. CFI > 0.95 = Excellent, > 0.90 = Good; RMSEA < 0.05 = Good, 0.05–0.10 = Moderate, >0.10 = Bad*.

**Table 2:** **Factor loadings for alternative models of the UCLA-3 Loneliness Scale, with variance explained (*R*^2^) for each item**.

	**1 Factor**		**2 Factor**			**3 Factor**			
***UCLA item: How often do you feel…***.	**Loneliness**	***R*^2^**	**Intimate**	**Social**	***R*^2^**	**Isolation**	**Relational connectedness**	**Collective connectedness**	***R*^2^**
U1: “In tune” with the people around you?	0.58	0.34		0.62	0.39			0.65	0.42
U2: That you lack companionship?	0.70	0.50	0.73		0.53	0.73			0.53
U3: That there is no one you can turn to?	0.82	0.67	0.86		0.73	0.86			0.73
U4: Alone?	0.74	0.55	0.76		0.58	0.76			0.58
U5: Feel part of a group of friends?	0.72	0.52		0.77	0.60			0.81	0.66
U6: That you have a lot in common with the people around you?	0.71	0.50		0.75	0.57			0.79	0.63
U7: That you are no longer close to anyone?	0.81	0.66	0.85		0.72	0.85			0.72
U8: That your interests and ideas are not shared by those around you?	0.67	0.44	0.61		0.48	0.69			0.48
U9: Outgoing and friendly?	0.57	0.32		0.60	0.36			0.63	0.40
U10: Close to people?	0.81	0.66		0.86	0.73		0.88		0.77
U11: Left out?	0.68	0.46	0.70		0.49	0.70			0.49
U12: That your relationships with others are not meaningful?	0.73	0.53	0.75		0.57	0.75			0.57
U13: That no one really knows you well?	0.77	0.60	0.80		0.64	0.80			0.64
U14: Isolated from others?	0.85	0.72	0.87		0.75	0.87			0.75
U15: You can find companionship when you want it?	0.60	0.36		0.64	0.41		0.65		0.42
U16: That there are people who really understand you?	0.69	0.48		0.74	0.55		0.75		0.57
U17: Shy?	0.46	0.21	0.48		0.23	0.48			0.23
U18: That people are around you but not with you?	0.73	0.54	0.76		0.58	0.76			0.58
U19: That there are people you can talk to?	0.85	0.72		0.89	0.78		0.90		0.80
U20: That there are people you can turn to?	0.86	0.74		0.90	0.81		0.91		0.83

*All loadings were significant, p < 0.05*.

### Phase 2: Confirming the factor structure of schizotypy

A preliminary analysis of the positive and negative schizotypy factors on their own, showed that two items (PAb Q6 and PAb Q8) had tetrachoric correlations of 1 with other items, therefore these were removed from subsequent analyses. Fit statistics for the 2 factor (positive and negative schizotypy) model were excellent, as shown in Table 1. Positive and negative schizotypy correlated 0.49, *p* < 0.001. Again, inspection of the indirect loadings revealed that none exceeded the direct loadings suggesting the model has discriminant validity (Thompson, 1997; see Supplementary Table 2 for pattern and structure matrices and *R*^2^ explained in each item).

### Phase 3: Do the loneliness and schizotypy sub-factors exist outside a general psychopathology factor?

**Figure 1:**
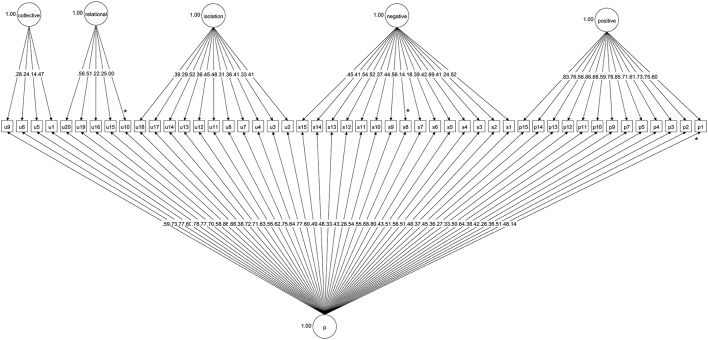
**Categorical bi-factor analysis involving a general psychopathology factor and five orthogonal sub-factors (three UCLA-3 loneliness factors, and two schizotypy factors)**. “p”, General Psychopathology Factor; WSS-B sub-factors: Positive = Positive Schizotypy, Negative = Negative Schizotypy; UCLA-3 Loneliness Scale sub-factors: isolation, relational = relational collectedness, collective = collective collectedness. ^*^Indicates non-significant paths in the bi-factor model.

Results of the confirmatory bi-factor analysis involving a general psychopathology or “p” factor and five orthogonal sub-factors (three UCLA-3 loneliness factors, and two schizotypy factors) are illustrated in Figure 1. The CFI, TLI, and RMSEA statistics all indicated that the model provided an excellent fit (see Table 1). The high and significant factor loadings (see Supplementary Table 3 for variance explained by each item) both for the general psychopathology factor and for the additional sub-factors suggests that it is useful to consider psychopathology both in terms of a unidimensional construct “p” and in terms of subfactors—each capturing separate variance. All but three items (SA Q8, PAB Q1, and UCLA Q10) loaded significantly on both the general “p” factor and the sub-factors.

### Phase 4: What is the pattern of inter-relationships between sub-factors of loneliness and schizotypy traits?

Zero order correlations between the three loneliness factors and SA were strong and significant (Isolation *rho* = 0.57, *p* < 0.01; Relational connectedness *rho* = 0.56, *p* < 0.01; Collective connectedness *rho* = 0.60, *p* < 0.01), whilst those with PAB were small and significant (Isolation *rho* = 0.27, *p* < 0.01; Relational connectedness *rho* = 0.21, *p* < 0.01; Collective connectedness *rho* = 0.17, *p* < 0.01). However, once the shared variance with the general psychopathology factor was removed, none of the correlations between the sub-factors of loneliness and the schizotypy subscales was significant, suggesting that they are independent constructs (Isolation with PAb: *r* = −0.10, *p* = 0.59; Isolation with SA: *r* = −0.16, *p* = 0.18; Relational connectedness with PAb: *r* = −0.17, *p* = 0.42; Relational connectedness with SA: *r* = 0.01, *p* = 0.89; Collective connectedness with PAb: *r* = −0.30, *p* = 0.24; Collective connectedness with SA: *r* = 0.15, *p* = 0.25).

## Discussion

The aim of the study reported here was to investigate the relationship between subjective feelings of loneliness and positive and negative trait schizotypy. The mean loneliness score was higher in the current sample of predominantly young adults compared to that previously reported in adolescents, aged 16–18 years, however, the spread of scores was similar (Lasgaard, 2007; Shevlin et al., 2014). Mean scores and score distributions for the PAb and SA scales were comparable to those previously reported in college students (Winterstein et al., 2011; Gross et al., 2012). The first phase of our analysis supported the multidimensional nature of loneliness assessed with the UCLA-3 Loneliness scale, with two and three-factor models providing acceptable fit to the data (Wilson et al., 1992; Hawkley et al., 2005). Increasing evidence demonstrates that the latter provides the best fit from adolescence to adulthood and generalizes across gender and culture (Hawkley et al., 2005, 2012; Shevlin et al., 2015), and the present findings suggest that this model—consisting of correlated facets of Isolated, Relational and Collective loneliness—extends to a predominantly young adult Australian sample. Consequently, we utilized the three-factor model in the remainder of our analyses.

The second phase of our analyses confirmed that a two-factor structure, of positive and negative schizotypy, provided a good fit to the current data, consistent with previous evidence (Gross et al., 2015). The final phases of our analyses suggest that loneliness and psychosis proneness, or schizotypy, are distinct and separable constructs, when measured in terms of positive (Perceptual Aberration) and negative (Social Anhedonia) schizotypy. Finally, our confirmatory bi-factor analysis provided strong support for a latent general factor of psychopathology, with high loadings on all (but one) of the individual items assessing loneliness and schizotypal traits. This finding is consistent with previous proposals that the structure of mental disorders is best characterized by a model which includes a common or general psychopathology factor “p,” in addition to more specific factors or dimensions (Caspi et al., 2014; Laceulle et al., 2015). Of particular importance here, when taking into account the existence of this latent general factor, the (three) loneliness and (two) schizotypy sub-factors remained, supporting the hypothesis that the three facets of loneliness and two facets of positive and negative schizotypal traits are separate constructs that explain a significant proportion of variance not accounted for by general psychopathology. Furthermore, the significant, positive correlations initially observed between isolation, related and collective connectedness and positive and negative schizotypy became non-significant once the shared variance with *p* was removed. Thus, while loneliness and schizotypy commonly co-occur and have previously been considered as reflecting the same construct (Gruzelier, 1996; Gross et al., 2012), the current findings suggest that they are distinct manifestations of psychological functioning. These results may be particularly important in view of recent evidence that schizotypal items assessing a lack of close interpersonal relations significantly predict transition to psychosis (Salokangas et al., 2013). The present study suggests that a sense of disconnection from others, i.e., loneliness, may in fact be a separate feature from trait schizotypy, with a different mechanistic influence on psychosis proneness. For example, Murphy and colleagues have argued that loneliness is a significant contextual vulnerability factor that can strengthen the relationship between negative childhood experiences from others and emergence of psychotic symptoms (Murphy et al., 2015).

Although loneliness is conceptually independent from schizotypy, the present findings suggest that there is overlap between the two constructs as part of a general psychopathology domain. This is not surprising, as previous research has shown that loneliness is associated with a range of personality “health” factors (i.e., emotional stability, surgency, agreeableness, conscientiousness, shyness, and sociability (Cacioppo et al., 2006), as well as depression, anxiety, and other psychopathological factors (Leary, 1990; Cacioppo and Cacioppo, 2014). Importantly, general psychopathology is an important construct for understanding schizophrenia-spectrum pathology and schizotypy, as negative affect/neuroticism, depression, and anxiety, are common in schizotypy (Horan et al., 2008; Hartley et al., 2013), and may serve as non-specific vulnerability markers or “state” markers of risk; potentially signaling onset of psychosis, relapse or decompensation more generally. Importantly, loneliness is commonly—but not universally—reported in people with, or vulnerable to, psychosis and is hypothesized to increase the risk of a range of mental illnesses, especially in adolescence (Lim and Gleeson, 2014; Shevlin et al., 2014; Badcock et al., 2015). Thus, feeling lonely may need to be understood both as a separate problem from schizotypy *and* as a marker of general psychopathology, rather than as an inherent feature of high schizotypy, requiring routine assessment and targeted intervention.

One limitation of the current study is that it focussed on investigating links between loneliness and perceptual aberration and social anhedonia, whilst schizotypy encompasses a broader range of traits. Factor analysis of schizotypy measures typically reveals 2–4 separate schizotypy factors (Badcock and Dragović, 2006; Fonseca-Pedrero et al., 2013; Gross et al., 2014). It is possible, therefore, that loneliness is specifically related to other facets of positive and negative schizotypy not assessed here, or to odd speech and behavior, associated with disorganized schizotypy. A second limitation is that the study samples were comprised of high functioning young adults enrolled at university, and therefore may not be representative of the general population. Consequently, replicating these results in a more diverse community sample would be useful. Similarly, the gender ratio in the current sample was uneven, the number of male participants was relatively small and separate analysis by sex was not undertaken. However, in future research it may be interesting to investigate whether the structure of the relationship between loneliness and schizotypy is consistent across sex. Finally, the measures employed in this study were exclusively self-report in nature and the methods of assessing random/careless responding differed at each study site, with unknown influence on the results. Although loneliness is an inherently subjective phenomenon, understanding its behavioral and neurobiological concomitants and how they relate to interviewer-based, or endophenotype-based measures of schizotypy is an important direction for future research.

## Author contributions

JB, EB, AC, and DB developed the idea for the study. RB conducted the confirmatory and bi-factor analyses and provided overall statistical advice. JB wrote the first draft of the manuscript. All authors helped with revisions to the manuscript and approved the final version of the article for submission.

### Conflict of interest statement

The authors declare that the research was conducted in the absence of any commercial or financial relationships that could be construed as a potential conflict of interest.

## Footnotes

^1^The SA scale was used at both study sites, but the PAb was not used at the UoW.

^2^Retrieved from: https://www.statmodel.com/wlscv.shtml
