# Supplementary material for: Loneliness and Schizotypy Are Distinct Constructs, Separate from General Psychopathology
Source: Front Psychol. 2016 Jul 7;7:1018. doi: 10.3389/fpsyg.2016.01018 (PMC4935680; doi:10.3389/fpsyg.2016.01018)
Supplement: Supplementary file 1 [file Table1.DOCX]

Supplementary Table 1.  Pattern and structure matrix for the UCLA-3 Loneliness Scale.

|  | 3 Factor | | | | | |
| --- | --- | --- | --- | --- | --- | --- |
|  | Relational connectedness with Isolation r = .777 | | | | | |
|  | Relational connectedness with Collective connectedness r = .863 | | | | | |
|  | Isolation with Collective connectedness r = .785 | | | | | |
| ***UCLA item: How often do you feel….*** | Isolation | | Relational connectedness | | Collective connectedness | |
|  | Pattern | Structure | Pattern | Structure | Pattern | Structure |
| U1: “In tune” with the people around you? | 0 | 0.51 | 0 | 0.51 | 0.65 | 0.65 |
| U2: That you lack companionship? | 0.73 | 0.73 | 0 | 0.57 | 0 | 0.57 |
| U3: That there is no one you can turn to? | 0.86 | 0.86 | 0 | 0.67 | 0 | 0.68 |
| U4: Alone? | 0.76 | 0.76 | 0 | 0.59 | 0 | 0.60 |
| U5: Feel part of a group of friends? | 0 | 0.64 | 0 | 0.70 | 0.81 | 0.81 |
| U6: That you have a lot in common with the people around you? | 0 | 0.62 | 0 | 0.68 | 0.79 | 0.79 |
| U7: That you are no longer close to anyone? | 0.85 | 0.85 | 0 | 0.66 | 0 | 0.67 |
| U8: That your interests and ideas are not shared by those around you? | 0.69 | 0.69 | 0 | 0.54 | 0 | 0.54 |
| U9: Outgoing and friendly? | 0 | 0.49 | 0 | 0.54 | 0.63 | 0.63 |
| U10: Close to people? | 0 | 0.68 | 0.88 | 0.88 | 0 | 0.76 |
| U11: Left out? | 0.7 | 0.70 | 0 | 0.54 | 0 | 0.55 |
| U12: That your relationships with others are not meaningful? | 0.75 | 0.75 | 0 | 0.58 | 0 | 0.59 |
| U13: That no one really knows you well? | 0.8 | 0.80 | 0 | 0.62 | 0 | 0.63 |
| U14: Isolated from others? | 0.87 | 0.87 | 0 | 0.68 | 0 | 0.68 |
| U15: You can find companionship when you want it? | 0 | 0.51 | 0.65 | 0.65 | 0 | 0.56 |
| U16: That there are people who really understand you? | 0 | 0.58 | 0.75 | 0.75 | 0 | 0.65 |
| U17: Shy? | 0.48 | 0.48 | 0 | 0.37 | 0 | 0.38 |
| U18: That people are around you but not with you? | 0.76 | 0.76 | 0 | 0.59 | 0 | 0.60 |
| U19: That there are people you can talk to? | 0 | 0.70 | 0.9 | 0.90 | 0 | 0.78 |
| U20: That there are people you can turn to? | 0 | 0.71 | 0.91 | 0.91 | 0 | 0.79 |

Supplementary Table 2. Pattern and Structure matrix for positive and negative schizotypy scales.

| Pattern and structure matrix for PAb and SA scales | 2 Factor  PAb with SA r = .493 | | | | |
| --- | --- | --- | --- | --- | --- |
|  | Positive | | Negative | |  |
|  | Pattern | Structure | Pattern | Structure | R^2^ |
| *Perceptual Aberration Scale* |  |  |  |  |  |
| P1: Occasionally it has seemed as if my body had taken on the appearance of another person’s body. | 0.54 | 0.54 | 0 | 0.26 | 0.29 |
| P2: I have sometimes felt confused as to whether my body was really my own | 0.89 | 0.89 | 0 | 0.44 | 0.79 |
| P3: I have sometimes had the feeling that my body is decaying inside. | 0.89 | 0.89 | 0 | 0.44 | 0.80 |
| P4: Sometimes I have felt that I could not distinguish my body from other objects around me. | 0.71 | 0.71 | 0 | 0.35 | 0.51 |
| P5: I have felt that something outside my body was a part of my body. | 0.74 | 0.74 | 0 | 0.37 | 0.55 |
| P7: Sometimes I have had a passing thought that some part of my body was rotting away. | 0.94 | 0.94 | 0 | 0.46 | 0.88 |
| P9: I can remember when it seemed as though one of my limbs took on an unusual shape. | 0.84 | 0.84 | 0 | 0.42 | 0.71 |
| P10: I sometimes have to touch myself to make sure I’m still there. | 0.88 | 0.88 | 0 | 0.43 | 0.78 |
| P11: I have sometimes had the feeling that one of my arms or legs is disconnected from the rest of my body. | 0.85 | 0.85 | 0 | 0.42 | 0.73 |
| P12: I have had the momentary feeling that my body has become misshapen. | 0.90 | 0.90 | 0 | 0.44 | 0.81 |
| P13: I have had the momentary feeling that my body has become misshapen. | 0.61 | 0.61 | 0 | 0.30 | 0.37 |
| P14: Parts of my body occasionally seem dead or unreal. | 0.85 | 0.85 | 0 | 0.42 | 0.72 |
| P15: At times I have wondered if my body was really my own. | 0.94 | 0.94 | 0 | 0.46 | 0.89 |
| *Social Anhedonia Scale* |  |  |  |  |  |
| S1: Having close friends is not as important as many people say | 0 | 0.30 | 0.61 | 0.61 | 0.37 |
| S2: I never had really close friends in high school. | 0 | 0.28 | 0.57 | 0.57 | 0.33 |
| S3: I prefer watching television to going out with other people. | 0 | 0.31 | 0.63 | 0.63 | 0.40 |
| S4: Just being with friends can make me feel really good. | 0 | 0.42 | 0.86 | 0.86 | 0.74 |
| S5: I’m much too independent to really get involved with other people. | 0 | 0.33 | 0.67 | 0.67 | 0.45 |
| S6: I prefer hobbies and leisure activities that do not involve other people. | 0 | 0.28 | 0.57 | 0.57 | 0.32 |
| S7: I don’t really feel very close to my friends. | 0 | 0.34 | 0.70 | 0.70 | 0.49 |
| S8: People who try to get to know me better usually give up after a while. | 0 | 0.33 | 0.67 | 0.67 | 0.45 |
| S9: Knowing that I have friends who care about me gives me a sense of security. | 0 | 0.38 | 0.77 | 0.77 | 0.60 |
| S10: People are usually better off if they stay aloof from emotional involvements with most others. | 0 | 0.35 | 0.71 | 0.71 | 0.50 |
| S11: If given the choice, I would much rather be with others than be alone. | 0 | 0.22 | 0.45 | 0.45 | 0.20 |
| S12: Although there are things that I enjoy doing by myself, I usually seem to have more fun when I do things with other people. | 0 | 0.33 | 0.66 | 0.66 | 0.44 |
| S13: I feel pleased and gratified as I learn more and more about the emotional life of my friends. | 0 | 0.29 | 0.58 | 0.58 | 0.34 |
| S14: When things are going really good for my close friends, it makes me feel good too. | 0 | 0.29 | 0.60 | 0.60 | 0.35 |
| S15: Making new friends isn’t worth the energy it takes. | 0 | 0.35 | 0.71 | 0.71 | 0.50 |

Supplementary Table 3. Factor loadings for the bifactor model comprising a general psychopathology factor, and 5 orthogonal subfactors, with variance explained (R^2^) for each item.

|  | General factor ‘p’ | Positive schizotypy | Negative schizotypy | Isolation | Relational  connectedness | Collective  connectedness | R^2^ |  |
| --- | --- | --- | --- | --- | --- | --- | --- | --- |
| *Social Anhedonia Scale* |  |  |  |  |  |  |  |  |
| S1: Having close friends is not as important as many people say | **0.37** |  | **0.52** |  |  |  | **0.40** |  |
| S2: I never had really close friends in high school. | **0.48** |  | **0.24** |  |  |  | **0.29** |  |
| S3: I prefer watching television to going out with other people. | **0.51** |  | **0.41** |  |  |  | **0.43** |  |
| S4: Just being with friends can make me feel really good. | **0.56** |  | **0.69** |  |  |  | **0.79** |  |
| S5: I’m much too independent to really get involved with other people. | **0.51** |  | **0.42** |  |  |  | **0.44** |  |
| S6: I prefer hobbies and leisure activities that do not involve other people. | **0.43** |  | **0.39** |  |  |  | **0.34** |  |
| S7: I don’t really feel very close to my friends. | **0.80** |  | **0.18** |  |  |  | **0.67** |  |
| S8: People who try to get to know me better usually give up after a while. | **0.68** |  | 0.14 |  |  |  | **0.49** |  |
| S9: Knowing that I have friends who care about me gives me a sense of security. | **0.55** |  | **0.57** |  |  |  | **0.62** |  |
| S10: People are usually better off if they stay aloof from emotional involvements with most others. | **0.54** |  | **0.44** |  |  |  | **0.48** |  |
| S11: If given the choice, I would much rather be with others than be alone. | **0.28** |  | **0.37** |  |  |  | **0.22** |  |
| S12: Although there are things that I enjoy doing by myself, I usually seem to have more fun when I do things with other people. | **0.44** |  | **0.52** |  |  |  | **0.46** |  |
| S13: I feel pleased and gratified as I learn more and more about the emotional life of my friends. | **0.33** |  | **0.54** |  |  |  | **0.40** |  |
| S14: When things are going really good for my close friends, it makes me feel good too. | **0.48** |  | **0.41** |  |  |  | **0.40** |  |
| S15: Making new friends isn’t worth the energy it takes. | **0.49** |  | **0.45** |  |  |  | **0.44** |  |
| *Perceptual Aberration Scale* |  |  |  |  |  |  |  |  |
| P1: Occasionally it has seemed as if my body had taken on the appearance of another person’s body. | 0.14 | **0.60** |  |  |  |  | **0.37** |  |
| P2: I have sometimes felt confused as to whether my body was really my own | **0.49** | **0.75** |  |  |  |  | **0.79** |  |
| P3: I have sometimes had the feeling that my body is decaying inside. | **0.51** | **0.73** |  |  |  |  | **0.80** |  |
| P4: Sometimes I have felt that I could not distinguish my body from other objects around me. | **0.36** | **0.61** |  |  |  |  | **0.50** |  |
| P5: I have felt that something outside my body was a part of my body. | **0.28** | **0.71** |  |  |  |  | **0.58** |  |
| P.7: Sometimes I have had a passing thought that some part of my body was rotting away. | **0.422** | **0.85** |  |  |  |  | **0.91** |  |
| P9: I can remember when it seemed as though one of my limbs took on an unusual shape. | **0.38** | **0.78** |  |  |  |  | **0.75** |  |
| P10: I sometimes have to touch myself to make sure I’m still there. | **0.64** | **0.59** |  |  |  |  | **0.76** |  |
| P11: I have sometimes had the feeling that one of my arms or legs is disconnected from the rest of my body | **0.50** | **0.68** |  |  |  |  | **0.71** |  |
| P12: I have had the momentary feeling that my body has become misshapen. | **0.33** | **0.86** |  |  |  |  | **0.85** |  |
| P13: Sometimes I feel like everything around me is tilting. | **0.27** | **0.56** |  |  |  |  | **0.38** |  |
| P14: Parts of my body occasionally seem dead or unreal. | **0.36** | **0.76** |  |  |  |  | **0.71** |  |
| P15: At times I have wondered if my body was really my own. | **0.45** | **0.83** |  |  |  |  | **0.88** |  |
| *UCLA-3 Loneliness Scale* |  |  |  |  |  |  |  |  |
| U1: “In tune” with the people around you? | **0.61** |  |  |  |  | **0.47** | **0.59** |  |
| U2: That you lack companionship? | **0.60** |  |  | **0.42** |  |  | **0.54** |  |
| U3: That there is no one you can turn to? | **0.77** |  |  | **0.33** |  |  | **0.70** |  |
| U4: Alone? | **0.64** |  |  | **0.41** |  |  | **0.57** |  |
| U5: Feel part of a group of friends? | **0.77** |  |  |  |  | **0.14** | **0.61** |  |
| U6: That you have a lot in common with the people around you? | **0.73** |  |  |  |  | **0.24** | **0.59** |  |
| U7: That you are no longer close to anyone? | **0.75** |  |  | **0.36** |  |  | **0.70** |  |
| U8: That your interests and ideas are not shared by those around you? | **0.62** |  |  | **0.31** |  |  | **0.48** |  |
| U9: Outgoing and friendly? | **0.59** |  |  |  |  | **0.28** | **0.42** |  |
| U10: Close to people? | **0.86** |  |  |  | 0.00 |  | **0.74** |  |
| U11: Left out? | **0.56** |  |  | **0.48** |  |  | **0.54** |  |
| U12: That your relationships with others are not meaningful? | **0.63** |  |  | **0.45** |  |  | **0.59** |  |
| U13: That no one really knows you well? | **0.71** |  |  | **0.36** |  |  | **0.63** |  |
| U14: Isolated from others? | **0.72** |  |  | **0.42** |  |  | **0.79** |  |
| U15: You can find companionship when you want it? | **0.59** |  |  |  | **0.25** |  | **0.40** |  |
| U16: That there are people who really understand you? | **0.70** |  |  |  | **0.22** |  | **0.54** |  |
| U17: Shy? | **0.38** |  |  |  |  |  | **0.23** |  |
| U18: That people are around you but not with you? | **0.66** |  |  |  |  |  | **0.59** |  |
| U19: That there are people you can talk to? | **0.77** |  |  |  | **0.51** |  | **0.85** |  |
| U20: That there are people you can turn to? | **0.78** |  |  |  | **0.56** |  | **0.91** |  |

*Note.* Items PABQ6 and PABQ8 were removed; Loadings in **bold** are significant, *p* < .05.
